# Structural analysis of pathogenic mutations in the *DYRK1A* gene in patients with developmental disorders

**DOI:** 10.1093/hmg/ddw409

**Published:** 2017-01-04

**Authors:** Jochem M.G. Evers, Roman A. Laskowski, Marta Bertolli, Jill Clayton-Smith, Charu Deshpande, Jacqueline Eason, Frances Elmslie, Frances Flinter, Carol Gardiner, Jane A. Hurst, Helen Kingston, Usha Kini, Anne K. Lampe, Derek Lim, Alison Male, Swati Naik, Michael J. Parker, Sue Price, Leema Robert, Ajoy Sarkar, Volker Straub, Geoff Woods, Janet M. Thornton, the DDD Study, Caroline F. Wright

**Affiliations:** 1 European Bioinformatics Institute (EMBL-EBI), Wellcome Genome Campus, Hinxton, Cambridge, UK; 2 Northern Genetics Service, Newcastle upon Tyne Hospitals NHS Foundation Trust, Institute of Human Genetics, International Centre for Life, Central Parkway, Newcastle upon Tyne, UK; 3 Manchester Centre for Genomic Medicine, St Marys Hospital, Central Manchester University Hospitals NHS Foundation Trust, Manchester Academic Health Science Centre, Manchester, USA; 4 Clinical Genetics Department, Guy’s and St Thomas’ NHS Foundation Trust, Guy’s Hospital, Great Maze Pond, London, UK; 5 Nottingham Regional Genetics Service, City Hospital Campus, Nottingham University Hospitals NHS Trust, The Gables, Hucknall Road, Nottingham, UK; 6 South West Thames Regional Genetics Centre, St George’s Healthcare NHS Trust, St George’s, University of London, Cranmer Terrace, London, UK; 7 West of Scotland Regional Genetics Service, NHS Greater Glasgow and Clyde, Institute Of Medical Genetics, Yorkhill Hospital, Glasgow, UK; 8 North East Thames Regional Genetics Service, Great Ormond Street Hospital for Children NHS Foundation Trust, Great Ormond Street Hospital, Great Ormond Street, London, UK; 9 Department of Clinical Genetics, Oxford University Hospitals NHS Foundation Trust, The Churchill Old Road, Oxford, UK; 10 South East of Scotland Clinical Genetics Service, Western General Hospital, Edinburgh, UK; 11 West Midlands Regional Genetics Service, Birmingham Women’s NHS Foundation Trust, Birmingham Women’s Hospital, Edgbaston, Birmingham, UK; 12 Sheffield Clinical Genetics Service, Sheffield Children's NHS Foundation Trust, Western Bank, Sheffield, UK; 13 East Anglian Medical Genetics Service, Box 134, Cambridge University Hospitals NHS Foundation Trust, Cambridge Biomedical Campus, Cambridge, UK and; 14 Wellcome Trust Sanger Institute, Wellcome Genome Campus, Hinxton, Cambridge, UK

## Abstract

Haploinsufficiency in *DYRK1A* is associated with a recognizable developmental syndrome, though the mechanism of action of pathogenic missense mutations is currently unclear. Here we present 19 *de novo* mutations in this gene, including five missense mutations, identified by the Deciphering Developmental Disorder study. Protein structural analysis reveals that the missense mutations are either close to the ATP or peptide binding-sites within the kinase domain, or are important for protein stability, suggesting they lead to a loss of the protein’s function mechanism. Furthermore, there is some correlation between the magnitude of the change and the severity of the resultant phenotype. A comparison of the distribution of the pathogenic mutations along the length of *DYRK1A* with that of natural variants, as found in the ExAC database, confirms that mutations in the N-terminal end of the kinase domain are more disruptive of protein function. In particular, pathogenic mutations occur in significantly closer proximity to the ATP and the substrate peptide than the natural variants. Overall, we suggest that *de novo* dominant mutations in *DYRK1A* account for nearly 0.5% of severe developmental disorders due to substantially reduced kinase function.

## Introduction

**Figure 1.**
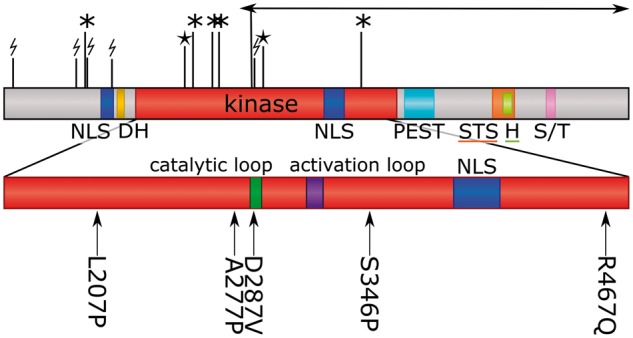
Domain arrangement of DYRK1A with location of diagnostic mutations. The kinase domain is enlarged and the catalytic loop and activation loop are labelled. NLS, nuclear localization signal; DH, DYRK homology box; PEST, proline, glutamic acid, serine, and threonine rich domain; STS, speckle-targeting signal; H, histidine repeat; S/T, serine/threonine repeat. Missense mutations are shown beneath the domain diagram, with position and amino acids; protein truncating variants are show above the diagram (* = stop-gained; lightning-bolt = frameshift; star = splice site; arrow = inversion). Arg437 to a stop codon occurred twice in the dataset.

The DYRK1A protein is a member of the highly conserved dual-specificity tyrosine-phosphorylation-regulated kinase (DYRK) family, which also includes the human members DYRK1B, DYRK2, DYRK3, and DYRK4 (1). Like all DYRK members, DYRK1A contains a conserved catalytic kinase domain preceded by a characteristic DYRK homology (DH) box, see Fig. 1. It also contains two nuclear localization signals (NLS), one prior to and one within the kinase domain, a PEST domain, a speckle-targeting signal (STS), a histidine repeat and a serine/threonine repeat (2). Although part of a Ser/Thr kinase family, the protein autophosphorylates the second tyrosine (Tyr321) of the activation loop YxY motif during translation. The tyrosine phosphorylation ability is lost once the protein is fully folded whereas the serine/threonine phosphorylation ability is retained (3–5).

The *DYRK1A* gene is located on chromosome 21 in the Down’s syndrome (DS) critical region, associated with the development of DS phenotypes when triplicated (6–10). In *Drosophila* the *DYRK1A* orthologue plays an essential role in neurogenesis, with mutant flies having a reduced brain size (1,11). Similarly, mice with only one functional copy of the gene have brains ∼30% smaller than those of wild type mice; moreover, mutations in mice also result in intrauterine growth restriction, behavioural defects and altered motor activity due to dopaminergic dysfunction (12–14). More recently, haploinsufficiency of *DYRK1A* in humans has been shown to cause intellectual disability, global developmental delay, microcephaly, intrauterine growth restriction, dysmorphic facial features, speech delay/absence, autism, febrile seizures, ocular malformations (15–27). Tejedor and Hammerle (10) reviewed the role of *DYRK1A* in neuronal development and characterized the protein as a regulator of a broad spectrum of neurodevelopmental mechanisms, listing many possible substrate and/or interacting proteins. A more recent study showed the protein is also recruited to promoters of genes actively transcribed by RNA polymerase II (RNAPII) after which it phosphorylates the C-terminal domain of RNAPII (28). Unfortunately, very little is known about its physiological substrate or interacting partners in neuronal development.

To date, only a handful of missense mutations in this gene have been associated with developmental phenotypes. The mechanism by which they cause disease is therefore unclear. Ji *et al.* analysed three missense mutations and observed that they occur in close proximity to the ATP binding site, which could account for their disruptive effect (26). Here we describe 19 new mutations, including five missense, as found in children in the Deciphering Developmental Disorder (DDD) Study (21), being children with previously undiagnosed severe developmental disorders. We analyse the locations of the missense mutations on the protein’s 3D structure to assess their likely impact on its stability and function. We also examine other mutations reported in the literature (29) and compare them with population variants obtained from the Exome Aggregation Consortium (ExAC) database (30).

## Results

**Figure 2.**
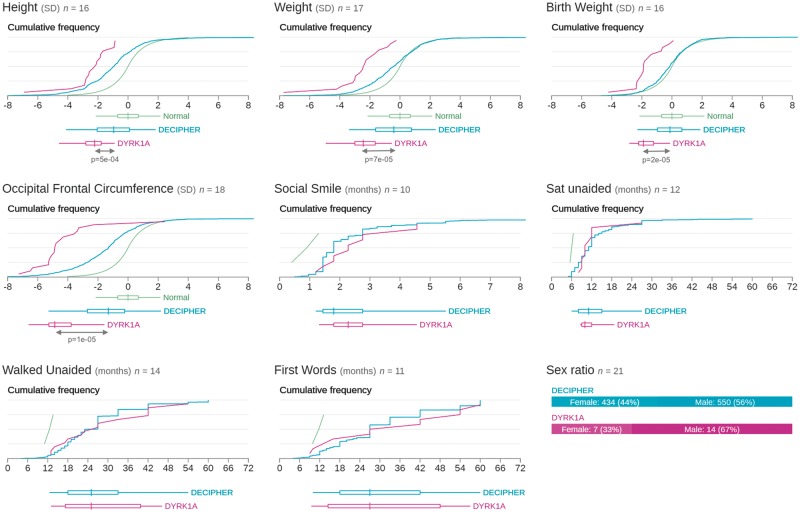
Phenicon comparing quantitative phenotypes for patients with variants in *DYRK1A* in DDD with all patients in DECIPHER and normal developmental parameters (An updated version is available online at https://decipher.sanger.ac.uk/gene/DYRK1A#overview/clinical-info). The *P*-values of all phenotypes with significant differences between the DYRK1A cohort and the DECIPHER cohort are shown below the plots.

An analysis of 4,293 family trios from the DDD study identified nineteen children as having likely pathogenic *de novo* mutations in *DYRK1A* (31). This corresponds to around 0.44% of those analysed (0.52% having intellectual disability). The mutations are listed in Table S1 with detailed patient phenotypes. Their locations are mapped onto the protein sequence in Fig. 1. All 19 patients have an intellectual disability and some dysmorphic facial features, 18 have microcephaly and restricted growth, 14 have eye malformations or visual impairments (including deeply set eyes, retinal dystrophy, optic atrophy, astigmatism, amblyopia, iris coloboma, bilateral microphthalmos, retinal detachment, hypermetropia and early cataracts), 11 have abnormal MRI scans, and 8 have seizures. A summary of quantitative phenotypes in these patients taken directly from the DECIPHER database (32) (https://decipher.sanger.ac.uk/gene/DYRK1A#overview/clinical-info) is shown in Fig. 2; one patient (DECIPHER ID 273659) is excluded as the database is unable to accept inversions at this time. Only one patient (DECIPHER ID 265726) does not have microcephaly, which is likely explained by an additional diagnostic *de novo* mutation in *TNNI2*, resulting in a complex compound phenotype.

**Figure 3.**
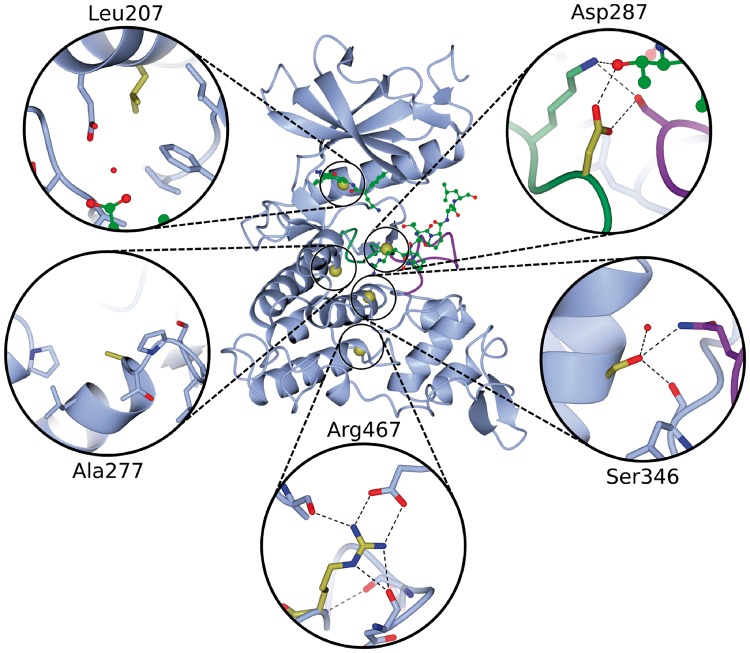
Ribbon diagram of the kinase domain of DYRK1A (PDB entry *2wo6*) with the Cα position of the five pathogenic missense mutations represented by gold spheres. The activation and catalytic loop are illustrated by the purple and green loops, respectively. The ATP and substrate peptide are depicted as green ball-and-stick models, where ATP is the smaller molecule. Each mutation has an inset to show the local environment of the mutated residue.

In our cohort, all 14 protein disrupting mutations (including six frameshift, two splice-site, five stop-gained and a 20kb intragenic inversion) occur before the C-terminal end of the kinase domain, so are likely to result in nonsense-mediated decay and complete loss of protein, rather than a truncated protein product. The five missense mutations all occur within the kinase domain, and their 3D locations are shown in Fig. 3. Each is described in turn below.

### The missense DDD mutations

The first is Leu207Pro which is located in an α-helix close to the ATP-binding pocket (Fig. 3). The replacement of this leucine by a proline will either break or kink the helix. Prolines are known as ‘helix breakers’ for two reasons: the side chain sterically interferes with the backbone of the preceding turn and the backbone nitrogen is unable to participate in backbone hydrogen bonds that stabilize α-helices. Moreover, according to the HSSP alignments, the leucine at this position is highly structurally conserved (99%); the only other amino acid observed at this position is the similar valine. Leu207 is highly buried – its accessible surface area (ASA) is 0.0, as calculated by the NACCESS program (33). This means it occupies a very specific space that a very different residue, such as a proline, would be incapable of filling. Hence it seems likely that the mutation will result in a conformational change affecting the ATP-binding pocket and disrupting the interactions necessary for the protein’s enzymatic activity. The patient with this mutation (DECIPHER ID 259211) has an intellectual disability with severe microcephaly (−4.3 SD) and growth restriction, as well as seizures, astigmatism and amblyopia, all in keeping with the haploinsufficiency syndrome previously described.

The second mutation, Ala277Pro, occurs on the surface of the protein immediately after an α-helix, in a loop in close proximity to the catalytic loop (Fig. 3). As for the first mutation above, the replacement by a proline is potentially problematic for the helix, although this residue is not strongly structurally conserved (13% alanine, 36% histidine, 11% lysine, 10% arginine). The entire loop that contains Ala277 is not structurally conserved, suggesting it is structurally and functionally of low importance. The φ backbone torsion angle of Ala277 is −81° whereas proline has a fixed φ-angle of around −65° (34) – so not too dissimilar. Thus, it is not clear why this mutation should result in the phenotype, since such a small change in the torsion angle could be compensated by minor angle changes in the rest of the loop. However, the backbone of the entire loop seems fairly stable. For example, in one of the highest-resolution structures of this kinase domain (PDB code 4ylk, solved at 1.4Å) the loop has a low ‘temperature-factor’ (between 8 and 13) together with a well-defined electron density. This indicates low flexibility. It is possible that the mutation disrupts the overall stability of the domain and, given its proximity to the catalytic loop, alters the structure around the catalytic loop, reducing its catalytic efficiency. In support of this, the patient (DECIPHER ID 267221) has an intellectual disability with microcephaly (−3.3 SD) and growth restriction, as well as delayed speech and language development and retinal dystrophy.

The deleterious effect of the third mutation, Asp287Val, is the most straightforward to explain as Asp287 is a catalytic residue directly involved in the reaction with the substrate; mutation to valine eliminates the catalytic ability of the protein. This amino acid is 100% conserved and also has important interactions with other conserved residues such as a hydrogen bond with Ser324 and a salt bridge to Lys289. Figure 3 shows the residue’s location and interactions. The affected patient (DECIPHER ID 258963) has an intellectual disability with severe microcephaly (−4.8 SD) and growth restriction, as well as delayed speech and language development and early cataracts.

The Ser346Pro mutation has previously been observed in two patients with a similar phenotype (15), although no structural analysis has been performed before. The residue is located in the middle of an α-helix and the side-chain forms three hydrogen bonds, one of which is with the side chain of Gln323 in the activation loop, see Fig. 3. This residue is highly structurally conserved (99% serine, 1% alanine), suggesting it plays an important role in the stability of the protein. Even though the backbone nitrogen does not form a hydrogen bond, replacement by a proline might, as in previous cases described above, be catastrophic for the stability of the α-helix. The patient (DECIPHER ID 260956) has an intellectual disability with very severe microcephaly (−7.3 SD) and growth restriction, as well as seizures.

Finally, the Arg467Gln mutation is the most distal pathogenic mutation in our cohort, far from the substrate and ATP-binding pocket, being in a loop near the C-terminal end of the kinase domain. The residue is part of a network of electrostatic interactions, as shown in Fig. 3, and is expected to play an important role in the overall stability of the protein. Only arginine is capable of forming all these interactions and is the only amino acid that can fit into this specific space. Furthermore, it is 100% structurally conserved. Changing it to glutamine would likely disrupt the stability of the protein’s fold, although possibly not the structure of the catalytic or ATP-binding sites. Thus, it might reduce the efficiency of the kinase rather than eliminating it completely. Accordingly, the patient (DECIPHER ID 2701740) appears to have the least severe phenotype of the cohort, with intellectual disability, mild microcephaly (−2.3 SD) and truncal obesity.

### Other DYRK1A mutations

**Figure 4.**
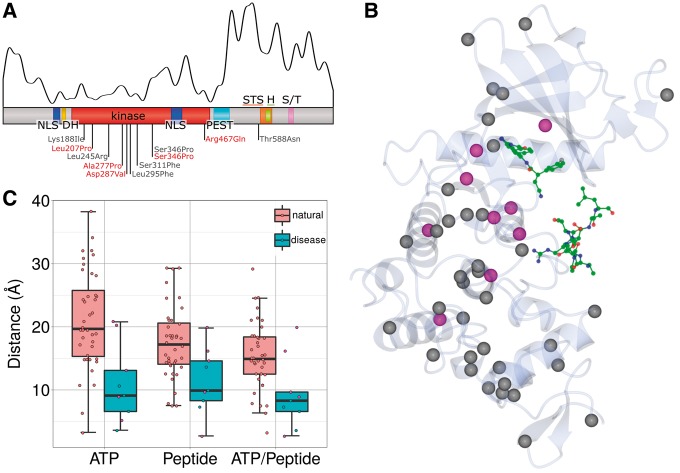
Analysis of pathogenic versus natural missense variations in DYRK1A. **(A)** Domain composition of DYRK1A (as in Figure 1) with the locations of the 171 natural variants from ExAC plotted as a smoothed curve above it. The locations of the known pathogenic missense mutations are labelled below the domain diagram, with the mutations from this study shown in red and mutations from the literature in grey (Ser346Pro occurs in both). **(B)** Ribbon diagram of the DYRK1A kinase domain (transparent), taken from PDB entry *2wo6*. The Cα position of pathogenic missense mutations shown as magenta sphere and natural variants as grey spheres. The green ball-and-stick models represent ATP (top) and the substrate peptide (bottom). **(C)** Boxplot of the distance between the mutated residues in DYRK1A and the ATP or the substrate peptide. ATP/Peptide gives the shortest distance to either the ATP or the peptide. The magenta filled points correspond to the five missense mutations from the DDD study.

We then compared the locations of the known missense pathogenic mutations in *DYRK1A* (five from this study and six from the literature (15,25,26)) on the 3D structure of the protein with the locations of natural, non-disease-associated variants listed in ExAC (Fig. 4). The gene is highly constrained, but the distributions of the two sets of variants are quite different, with most of the natural ones found either prior to the first nuclear localization signal or after the kinase domain (Fig. 4A). The natural variants in the kinase domain are mostly located at the C-terminal end whereas most of the pathogenic mutations occur in the N-terminal half, supporting the notion that the mutational burden is overall higher in this region. Figure 4B shows the 3D locations of the pathogenic mutations (magenta) and the population variants (grey). The former cluster around the ATP and peptide, whereas the latter tend to lie further away, and particularly in the C-terminal (lower) part of the domain. The boxplot in Fig. 4C shows the closest distances of each of the variants from either the ATP or the bound peptide, or from one or the other. In all three cases, the disease-causing mutations are significantly closer to the bound molecule than the population variants (Wilcoxon-Mann-Whitney test: *P =* 0.001, *P =* 0.01 and *P =* 0.008 for ATP, peptide and the closest of the two, respectively).

The mutation effect prediction program SIFT (35) predicted all disease-associated mutations within the kinase domain to be deleterious but predicted the Thr588Asn mutation from a previous study (which lies outside the domain) to be non-deleterious. Of the 171 natural variants, 112 were predicted to be non-deleterious, 32 to be damaging with a low confidence interval and 27 were predicted to be deleterious of which 21 occurred in the kinase domain. These results underline the importance of the kinase domain.

## Discussion

We have described 19 pathogenic *de novo* dominant mutations in *DYRK1A*, taking the total number of mutations described in the literature to over 70. Pathogenic sequence mutations in this gene result in loss of protein function and account for around 0.5% of syndromic intellectual disability. Patients typically have global developmental delay and microcephaly (average head circumference in our cohort = −4.6 SD) with a number of other common phenotypes including delayed speech and language, growth restriction, dysmorphic facial features, eye malformations and seizures. The phenotypes and molecular mechanisms described here are consistent with haploinsufficiency.

Protein structural analysis of the missense mutations in our cohort indicates that the affected residues are crucial for catalytic function or stability of the DYRK1A kinase domain. The phenotypic impact of some of the missense mutations appears as severe as that of the loss-of-function mutations, suggesting they may be disrupting the protein’s function as comprehensively as the loss-of-function cases. Furthermore, there does appear to be some genotype-phenotype correlation, in that the more severe phenotypes are seen when the missense variant is closest to the catalytic loop or either of the substrate binding sites. Analysis of the distribution of variation within the domain structure of the protein is also informative with respect to other mutations. Interestingly, while all the pathogenic splice site mutations identified in this study occur within the kinase domain itself, which is likely to be intolerant to alternative splicing, likely benign splice variants and indels occur either proximal to the start of the N-terminus of the domain or distal to the C-terminal end where alternatively spliced isoforms of the protein may be viable. Similarly, benign in-frame insertions/deletions both within ExAC and the DDD dataset also occur distal to the kinase domain, where the addition or removal of amino acids is unlikely to substantially alter the catalytic efficiency of the protein. *In vitro* experiments could be performed in future work to elucidate the effects of natural variants on the protein compared to the pathogenic variants. It would be interesting to investigate whether these findings in DYRK1A hold true for other kinases involved in disease.

## Materials and methods

The DDD study was approved by the UK Research Ethics Committee (10/H0305/83, granted by the Cambridge South REC, and GEN/284/12 granted by the Republic of Ireland REC), and appropriate informed consent was obtained from all participants. Patients meeting the recruitment criteria (neurodevelopmental disorder and/or congenital anomalies, abnormal growth parameters, dysmorphic features and unusual behavioural phenotypes) were recruited to the DDD study (www.ddduk.org) by their UK NHS and Republic of Ireland Regional Genetics Service, who also recorded clinical information and phenotypes using the Human Phenotype Ontology (36) via a secure web portal within the DECIPHER database (32). DNA samples from patients and their parents were analysed by the Wellcome Trust Sanger Institute using high-resolution microarray analysis (array-CGH and SNP-genotyping) to investigate copy number variations in the child and by exome sequencing to investigate single nucleotide variants and small insertions/deletions (indels). All genomic variants were annotated with the most severe consequence predicted by Ensembl Variant Effect Predictor (37) and their minor allele frequencies observed in diverse population samples. As has been described previously (38), likely diagnostic variants were communicated to referring clinical geneticists for validation in an accredited diagnostic laboratory and discussion with the family via patients’ record in DECIPHER, where they can be viewed in an interactive genome browser.

Several Protein Data Bank (PDB) structures of the DYRK1A protein are available, all limited to the DH-box and kinase domain (residues 137–479 of the protein). Here, we use PDB accession *4ylk* (39) to study the structural locations of the mutated residues. It has the highest resolution and has been published most recently of all the DYRK1A structures. We also used PDB accession *2wo6* (40), since this structure contains a bound substrate peptide. The structures were analysed and figures were made using CCP4mg (41). Structural conservation was extracted from the HSSP (homology-derived structures of proteins) database (42), which uses 194 sequences predicted to be structurally similar to PDB accession *4ylk*. Population variants, 176 in total (171 missense), were retrieved from Exome Aggregation Consortium (ExAC), Cambridge, MA (URL: http://exac.broadinstitute.org) [date (April, 2016) accessed]. Because none of the available PDB structures of DYRK1A is in complex with ATP, the distance between a mutated residue and the bound ATP was estimated by taking the distance between the residue and the ATP inhibitor. Distances were calculated by taking the shortest atom-atom distance between the residue and the molecule of interest (the latter being either ATP or the substrate peptide). The shortest distance to both ATP and substrate peptide was also calculated.

## Supplementary Material

Supplementary Material is available at *HMG* online.

## Supplementary Material

Supplementary Data
